# American Institute, Session of 1890

**Published:** 1890-05

**Authors:** Pemberton Dudley

**Affiliations:** General Secretary, S. W. Cor. 15th and Master Streets, Philadelphia


### THE AMERICAN INSTITUTE, SESSION OF 1890.

EDITOR HOMŒOPATHIC PHYSICIAN:

As already announced by circular to the members of the American Institute of Homœopathy, the next annual session of this body will be held at "Fountain Spring House," Waukesha, Wisconsin, commencing at 7.30 P. M., Monday, June 16th, and closing Friday, June 20th, 1890.

Waukesha, "The Saratoga of the West," famous for its "Bethesda," "Silurian," "Fountain," "Clysmic," and other mineral springs, is a town of six thousand inhabitants, situated about one hundred miles north of Chicago, and twenty miles west of Milwaukee, and directly on important lines of railroad. The hotel in which the session will be held is an immense stone and brick structure, capable of accommodating eight hundred guests, and furnished with all modern conveniences. It is situated in a beautiful park of one hundred and fifty-five acres, laid out with drives, shaded walks, flower gardens, etc., while the town itself presents numerous attractions to visitors in search of either health or pleasure.

The local Committee of Arrangements is making provision for the comfort and enjoyment of those who may attend the session, such as to render the occasion one of the most memorable in the Institute's history.

Under the new rule, the Bureaus will present a far greater variety of subjects for discussion than heretofore, and the papers will embrace more of the observation and experience of their writers. Important subjects of professional interest will be introduced and acted upon, and interesting reports will be presented by several committees.

Any paper, after being presented at the session, may be published in the journals at the discretion of its writer. With a view to such outside publication, writers are especially requested to have their papers *in duplicate.*

Officers of homœopathic societies and institutions are urged to make prompt reports (on blanks which will shortly be sent to them) to the Bureau of Organization, Registration, and Statistics.

All hospitals and dispensaries, so reporting, will receive a pamphlet copy of the entire Statistical Report of the Institute.

It is desirable that the Institute should receive this year another large addition to its membership, particularly from the West and Northwest, in order to secure a more equal apportionment of membership as between the East and the West, and to augment the influence of our school in shaping legislation and defending the equal rights of homœopathists in public institutions, appointments, etc. It has been suggested that each State and local society should provide for a complete canvass of its membership in order to secure for itself a larger representation in the membership of the National Society. The initiation fee is two dollars, annual dues, five dollars, entitling the member to the annual volume of *Transactions.* Blank applications for membership can be obtained of the undersigned.

The Annual Circular, giving full details of the session, the programme, railroad fares, hotel rates, etc., will be issued in May. Any physician failing to receive a copy by May 25th, can obtain one on application to

PEMBERTON DUDLEY, *General Secretary,*

S. W. Cor. 15th and Master Streets, Philadelphia.

